# A Few Observations

**Published:** 1899-02

**Authors:** R. B. Cummins

**Affiliations:** Blairsville, Pa.


ARTICLE V.

### A FEW OBSERVATIONS.

R. B. CUMMINS, D. D. S., BLAIRSVILLE, PA.

Scientific experiments are a great thing, but how often they are hobbies ridden to death.

Antisepsis is one the medical and dental professions are exercising now.

Only a few days ago we heard of a mother who was trying to follow out the instructions of the family physician. She said they were about to give up trying to give their son an education as they could not get him sterilized every morning in time to go to school.

It is pleasing to think of the amount of common seuse advice that can be given by the dentist at the chair, and of what good fruit it will bear. But if we get into the microbe war and give a long worded wearisome talk of their action on matter, molecules, the patient will begin to wonder how long it takes to fill a tooth anyway.

We must not shirk our duty in respect to teaching patients how to take care of the work or services we have rendered them, as upon their treatment of it depends very much of its permanence. Jonah tried to shirk his work, and remember what came of him.

Teeth may be preserved if we do our work well and the patient takes care of it. Dentist and patient must work together or there will be lots of failures and the former will get all to his credit.

Tell patients to brush their teeth after every meal. If they cannot brush teeth but once a day, tell them to do so just before retiring.

Encourage patients to learn the value of their teeth and they will appreciate and care for them. Tell the patients who use their influence for you that they cannot do a better work for you than to take good care of the fillings you have inserted for them.

When, by a united effort, we can frame a law that will be in accord with the ideas of every State dental society in the Union, we may expect to show to the world that we stand, as one man, for what is right and lawful. Any practitioner who has conducted an honorable practice in one State should be permitted to remove to another, without having to undergo the ordeal of an examination under a board of dental examiners, who do not consider common sense one of the most necessary elements in the conducting of a dental practice.

Sydney Smith says, "Let every man be occupied, and occupied in the highest employment of which his nature is capable, and die with the consciousness that he has done his best."

Work is one of the best educators of character. It gives discipline, self-control, deftness and skill in his special calling. Work is the law of our being and gives aptitude and dexterity in dealing with the affairs of ordinary life. Hobbies are useful as educators of the working faculty. Hobbies bring industry of a certain kind, and at least provide agreeable occupation. It must not be ridden too hard, else, instead of recreating, refreshing and invigorating a man's nature, it may only have the effect of sending him back to his business exhausted and depressed. It is not work, but overwork that is hurtful; and it is not hard work that is injurious so much as monotonous work, fagging work, hopeless work.—*Ohio Journal.*

